# Fatty acid composition and metabolic partitioning of α-linolenic acid are contingent on life stage in human CD3^+^ T lymphocytes

**DOI:** 10.3389/fimmu.2022.1079642

**Published:** 2022-12-13

**Authors:** Annette L. West, Johanna von Gerichten, Nicola A. Irvine, Elizabeth A. Miles, Karen A. Lillycrop, Philip C. Calder, Barbara A. Fielding, Graham C. Burdge

**Affiliations:** ^1^ School of Human Development and Health, Faculty of Medicine, University of Southampton, Southampton, Hampshire, United Kingdom; ^2^ Department of Nutritional Sciences, Faculty of Health and Medical Sciences, University of Surrey, Guildford, Surrey, United Kingdom; ^3^ Centre for Biological Sciences, Faculty of Natural and Environmental Sciences, University of Southampton, Southampton, Hampshire, United Kingdom; ^4^ National Institute for Health and Care Research (NIHR) Southampton Biomedical Research Centre, University Hospital Southampton National Health Service (NHS) Foundation Trust and University of Southampton, Southampton, Hampshire, United Kingdom

**Keywords:** T lymphocyte, essential fatty acid, α-linolenic acid, hydroxyoctadecadienoic acids, life course, lipid droplet, CD69, fatty acid (composition)

## Abstract

**Introduction:**

Immune function changes across the life course; the fetal immune system is characterised by tolerance while that of seniors is less able to respond effectively to antigens and is more pro-inflammatory than in younger adults. Lipids are involved centrally in immune function but there is limited information about how T cell lipid metabolism changes during the life course.

**Methods and Results:**

We investigated whether life stage alters fatty acid composition, lipid droplet content and α-linolenic acid (18:3ω-3) metabolism in human fetal CD3^+^ T lymphocytes and in CD3^+^ T lymphocytes from adults (median 41 years) and seniors (median 70 years). Quiescent fetal T cells had higher saturated (SFA), monounsaturated fatty acid (MUFA), and ω-6 polyunsaturated fatty acid (PUFA) contents than adults or seniors. Activation-induced changes in fatty acid composition differed between life stages. The principal metabolic fates of [^13^C]18:3ω-3 were constitutive hydroxyoctadecatrienoic acid synthesis and β-oxidation and carbon recycling into SFA and MUFA. These processes declined progressively across the life course. Longer chain ω-3 PUFA synthesis was a relatively minor metabolic fate of 18:3ω-3 at all life stages. Fetal and adult T lymphocytes had similar lipid droplet contents, which were lower than in T cells from seniors. Variation in the lipid droplet content of adult T cells accounted for 62% of the variation in mitogen-induced CD69 expression, but there was no significant relationship in fetal cells or lymphocytes from seniors.

**Discussion:**

Together these findings show that fatty acid metabolism in human T lymphocytes changes across the life course in a manner that may facilitate the adaptation of immune function to different life stages.

## Introduction

Lipids, in particular polyunsaturated fatty acids (PUFAs), play critical roles in T lymphocyte function and differentiation (1) by maintaining the homeoviscosity of cell membranes (2, 3) and acting as substrates for the synthesis of lipid second messengers including oxylipins such as eicosanoids (4–10), hydroxyoctadecadienoic (HODEs) acid, hydroxyoctadecatrienoic acids (HOTrEs) (11–15), and specialised pro-resolving mediators (16, 17) as well as diacylglycerol and phosphatidic acid (18). Fatty acid oxidation and synthesis are required for regulating the differentiation of regulatory T cells (T_Reg_) (19).

Leukocytes, including T lymphocytes, can obtain fatty acids including PUFAs from their environment by a CD36-dependent mechanism that is up-regulated in activated cells, and which facilitates T_Reg_ survival and anti-tumour activity (20), but does not appear to discriminate between fatty acids.

There is some evidence that human immune cells can synthesise a limited range of PUFA species from their essential fatty acid (EFA) precursors, namely α-linolenic acid (18:3ω-3) and linoleic acid (18:2ω-6), by a modification of the pathway described in rat liver (21–23) in which the first reaction is the addition of 2 carbon atoms to 18:2ω-6 or 18:3ω-3 (24, 25) which is probably catalysed by elongase 5 (21–23). Extension of 18:2ω-6 and 18:3ω-3 to their 20 carbon elongation products in human peripheral blood mononuclear cells (PBMCs) and T lymphocytes is followed by desaturation at the Δ8 position, probably by the protein product of the *FADS2* gene, known as Δ6 desaturase (22, 23), and then desaturation at the Δ5 position by Δ5 desaturase. Human T lymphocytes do not appear to synthesise PUFAs with a chain length greater than 22 carbons (22, 23), because they do not express elongase 2 which in the liver is responsible for chain elongation of PUFA with 22 carbon atoms to 24 carbon PUFAs (24). Inhibition of the protein product of the *FADS2* gene, which catalyses the first desaturation reaction, can inhibit the proliferation of CD3^+^ T cells, although the underlying mechanism is not known (23). Moreover, partitioning of 18:2ω-6 and 18:3ω-3 between carbon chain elongation and synthesis of their 15-lipoxygenase metabolites namely HODEs or HOTrEs, respectively, has been suggested to be a putative metabolic branchpoint in EFA metabolism in human CD3^+^ T lymphocytes that can be modified by the ratio of the substrates 18:2ω-6 to 18:3ω-3 (22).

Cytoplasmic lipid droplets are dynamic specialised lipid structures central to the integration of immune function and metabolism which serve to provide an energy reserve and substrates for synthesis of lipid mediators and to ameliorate the cytotoxic effects of intracellular unesterified fatty acids and inappropriate activation of protein kinase C by cytoplasmic diacylglycerol (1, 25, 26). Accumulation of these neutral lipid inclusions has been inversely related to the proliferation of CD8^+^ T cells from seniors (27) and so altered metabolism of lipid droplets may represent one mechanism for impaired immune function in ageing.

Different life stages have characteristic patterns of immune function (28–30). For example, the fetal immune system has a greater tendency for tolerance than that of adults, due, at least in part, to a higher propensity to produce T_Reg_ cells (28). Such immune tolerance is essential for the survival of the fetus in the maternal environment as well as for maintaining immune homeostasis in the presence of foreign antigens such as those from the developing microbiota (28). In contrast, immune ageing is associated with a decline in the capacity to mount an acquired immune response to novel pathogens and vaccines (29, 30) which is accompanied by an increased tendency for inflammation (29, 30) that underlies a number of age-related non-communicable diseases (31).

Since T lymphocyte function differs between life stages, we hypothesised that T cells from humans at different life stages would differ in lipid composition and EFA metabolism. To test this, we analysed the fatty acid compositions of quiescent and mitogen-stimulated human CD3^+^ T lymphocytes isolated from umbilical cord blood and from the blood of healthy adults and older men and women (seniors), and measured the partitioning of [^13^C]18:3ω-3 between synthesis of longer chain PUFAs, HOTrEs and 𝛽-oxidation. We also determined the lipid droplet content of these cells.

## Materials and methods

### Ethics statement

The study was reviewed and approved by the East of England - Cambridge Central Research Ethics Committee (approval number 19/EE/0096) and all participants gave written informed consent. The purchase and use of adult and fetal primary leukocytes that were collected in accordance with Local, State and Federal U.S. requirements by StemCell Technologies, (Vancouver BC, Canada), was reviewed and approved by the University of Southampton Faculty of Medicine Ethics Review Committee (submission I.D.S 49658 and 58050.A1).

### Adult participants and collection of blood samples

The inclusion and exclusion criteria used to select participants were described previously (22). Briefly, adult participants were healthy men and women with a median age of 41 (range 21 – 48) years (n = 10 (4 women)). Seniors were healthy men and women with a median age 70 (range 58 – 74) years (n = 7 (4 women)) with median body mass index of 25.6 (24.1 – 26.5) kg/m^2^ and 26.7 (20.5 – 30.0) kg/m^2^, respectively. All participants had a blood pressure within age-adjusted normal ranges, non-fasting total cholesterol concentration < 7.5 mmol/L, HbA1c concentration < 42 mmol/mol, and a C-reactive protein concentration < 3 mg/L. Participants did not habitually consume fish oil or other dietary oil supplements, smoke tobacco or report any chronic disease, and were willing to follow the study protocol and able to provide written informed consent. Volunteers were excluded if they did not meet the inclusion criteria, were pregnant or intending to become pregnant during the study, or were already participating in a clinical trial.

Non-fasting venous blood samples from adult participants were collected into tubes containing lithium heparin anticoagulant on three occasions (100 mL each collection) separated by an interval of at least 4 weeks and from participants in the seniors group on one occasion (50 mL).

### Processing of commercially-sourced T lymphocytes and peripheral blood mononuclear cells

The targets for recruitment of participants and sample collection were not met completely because of the implementation of restrictions on population movement and social interactions by the United Kingdom Government in March 2020 in response to the SARS-CoV-2 pandemic (March 2020 onwards). The shortfall in adult samples was addressed by purchasing cryopreserved adult CD3^+^ T lymphocytes (Catalog number 70024.1) from StemCell Technologies UK Ltd (Cambridge, UK) that were collected from anonymous participants whose recorded characteristics met the inclusion criteria for the study. Umbilical cord peripheral blood mononuclear cells (PBMCs) from clinically normal pregnancies were purchased from StemCell Technologies (catalogue number 70007-C). Fetal CD3^+^ T lymphocytes were isolated by the same method as adult T cells. screened for HIV, hepatitis B and hepatitis C infection by StemCell Technologies UK ltd.).

### Isolation and culture of CD3^+^ T cells

CD3^+^ T cells were isolated from blood as described elsewhere (22). Briefly, whole blood from adults was layered onto histopaque and separated by centrifugation at 845 x g for 15 minutes at room temperature. PBMCs were collected by aspiration, diluted with an equal volume of RPMI 1640 medium containing 10% (v/v) homologous pooled heat-inactivated serum (Sigma-Aldrich, Dorset, UK) (Complete Medium). CD3^+^ T cells were isolated from PBMCs prepared from blood donated by adults and seniors and from purchased umbilical cord PBMCs by negative selection using the T cell EasySep kit (StemCell Technologies) according to the manufacturer’s instructions. After washing with 10 ml Complete Medium, isolated T cells were collected by centrifugation at 300 x g for 10 minutes at room temperature and cryopreserved (22, 25, 32–35). The CD3^+^ T lymphocyte content of the T cell preparations was typically fetal 94%, and adults and seniors 99%.

T lymphocyte culture was carried out as described elsewhere (22, 23). Briefly, cells were thawed and resuspended in RPMI1640 Complete Medium (22). The EFA composition of the medium was adjusted by the addition of 18:3ω-3 (final total concentration 40µmol/L including [1−^13^C]18:3ω-3 (4 µmol/L) (22) to give a 18:2ω-6: 18:3ω-3 ratio of 5:1 (including fatty acids present in the serum supplement) which favours conversion of 18:3ω-3 to longer chain PUFAs (22). The fatty acid composition of the medium was confirmed routinely by gas chromatography (GC). T cell cultures (1 × 10^6^ T cells/mL) were maintained in a humidified incubator at 37°C in an atmosphere containing 5% (v/v) CO_2_ for up to 48 hours with or without the addition of concanavalin A (10 μg/mL; Con. A; Sigma-Aldrich) (22). Cells were collected by centrifugation, washed with phosphate-buffered saline (PBS) and stored at −80°C or used immediately for flow cytometry. The cell density was determined by collecting an aliquot of the culture (20µL) at the start and end of the culture period the number of cells was determined using a Beckman Coulter Counter Z series-1.

### Analysis of T cell fatty acid composition by gas chromatography

CD3^+^ T cells were thawed and suspended in 0.9% (wt/v) NaCl. Heptadecanoic acid (3 µg) internal standard was added and total T cell lipids were extracted with chloroform/methanol (2:1, v/v) containing butyrated hydroxytoluene (50 mg/ml) (36, 37). Fatty acid methyl esters (FAMEs) were synthesised by incubation with methanol containing 2% (v/v) H_2_SO_4_ at 50°C for 120 minutes (37). The reaction mixture was cooled to room temperature, neutralised and FAMEs were collected by hexane extraction (37). FAMEs were resolved on a BPX-70 fused silica capillary column (30 m × 0.25 mm × 25 μm) using an Agilent 6890 gas chromatograph (Agilent, Cheshire, UK) and equipped with a flame ionisation detection (FID) (22, 38). FAMEs (2 μL) were injected in split mode *via* a split/splitless injection port held at 300°C with He carrier gas flow rate of 1mL min^-1^ (38). The initial oven temperature was held at 115°C for 2 min after injection, increased at 10°C min^-1^ to 200°C and held at this temperature for 16 min. The oven temperature was then increased at 60°C min^-1^ to 240°C and held for 2 min. The detector temperature was maintained at 300°C. Chromatograms were integrated manually by a single operator using ChemStation software (version B.03.01, Agilent Technologies). FAMEs were quantified by dividing the peak area of a target fatty acid by that of the internal standard, multiplied by the amount of internal standard added, and adjusted for the number of cells in the culture. The total amount of cell fatty acids was calculated from the sum of all fatty acids that were measured. The cell density was determined by collecting aliquots of the culture (20µL) at the start and and end of the culture period using a Beckman Z series coulter counter.

Fatty acids were identified by their retention times relative to standards (37 FAMES, Sigma-Aldrich) and their identities were confirmed *ad hoc* by GC-mass spectrometry (22) **Supplementary Figure 1**).

### Measurement of stable isotope-labelled fatty acids

[^13^C]-Enrichment of ω-3 PUFAs was measured by GC-combustion–isotope ratio mass spectrometry (22, 34). FAMEs were resolved using a Supelcowax 10 capillary column (30 m x 0.25 mm x 0.25 µm; Sigma-Aldrich, Dorset, UK) (He carrier flow rate 1.5 ml min^-1^) on a Thermo Trace 1310 gas chromatograph (ThermoFisher, Loughborough, UK) equipped with a high-temperature combustion furnace (1,000°C) and a Thermo Delta V isotope ratio mass spectrometer. The ^13^C/^12^C ratio was measured relative to laboratory reference gas standards that were calibrated against the Vienna Pee Dee Belemnite international standard and used to calculate fatty acid [^13^C] enrichment (22, 34). The quantity of each labelled fatty acid was calculated from the amount measured by gas chromatography with FID, normalised to the number of cells in the culture (22).

### Measurement of [^13^C]-labelled oxylipins in T cell culture supernatants

Stable isotope enrichment of [^13^C]18:3ω-3-derived oxylipins was determined by liquid chromatography-tandem mass spectrometry (LC-MS/MS) (22, 35). Culture supernatants were collected, centrifuged to remove any remaining cells and immediately frozen at -80°C. Butyrated hydroxytoluene and EDTA (both 0.2 mg/mL), indomethacin (100 µM) and 4-[[trans-4-[[(tricyclo[3.3.1.13,7]dec-1-ylamino)carbonyl]am ino]cyclohexyl]oxy]-benzoic acid (Cayman Chemicals, Cambridge U.K., catalogue number 16568) (100 µM) in methanol/water 1:1 (v/v) (40 µL) were added to prevent autooxidation of oxylipins (39) together with the internal standard [d_5_](17(*S*)-hydroxydocosa-4,7,10,13,15,19-hexaenoic-21,21,22,22,22-d5-acid ([d_5_]17-HDHA) (Cayman chemicals (catalogue number Cay29797-25, Cambridge, U.K.) (20 g x 10^-9^). The frozen supernatants (2.0 mL) were thawed at 4°C overnight. Proteins were precipitated with 750 µL ice cold methanol for 30 min at -20°C, acidified with 1M HCl (10 µL) and oxylipins were isolated by solid phase extraction using Oasis HLB (Waters) solid phase extraction cartridges (22, 39) and stored in 100 µL methanol/water 70:30 (v/v) at -20 °C and analysed within 24 hours by LC-MS/MS.

Oxylipins were analysed with multiple reaction monitoring (MRM) using an Acquity I-class and Xevo TQS UPLC-MS/MS system (Waters). Negative ESI parameters were: 2.4 kV capillary voltage, 40 V cone voltage, 600°C desolvation temperature, 1000 L h^-1^ desolvation flow, 150 L h^-1^ cone flow and 7 bar nebuliser pressure. MRM transitions measured were as described (22). Lipids were separated using a Cortecs C18 (2.1 mm x 100 mm, 1.6 µm) column (Waters) with a BEH C18 VanGuard (2.1 mm x 5 mm, 1.7 µm) pre-column (Waters) at 40°C with the autosampler temperature set at 10°C and a flow rate of 0.3 mL min^-1^. The linear gradient was run with mobile phase A (80:20 (v/v) water/acetonitrile) and mobile phase B (75:25 (v/v) acetonitrile/methanol), both containing 0.02% (v/v) formic acid starting at 20% mobile phase B for 1 min increasing to 35% B over 2 min and then 70% B for 7 min followed by 95% B for 2 min, held for 2 min, then returning to the initial solvent conditions (22).

The limits of detection, solid phase extraction recovery and quality control coefficient of variation were as described elsewhere (22). Data were processed using MassLynx 4.0 (Waters). Oxylipin concentrations were calculated relative to the internal standard [d_5_]17-HDHA, normalised to the supernatant volume, and the background enrichment corrected against media without additional EFA, and then normalised to the number of T cells in the cell culture (22).

### Measurement of T lymphocyte lipid droplet content and CD69 expression by flow cytometry

The lipid droplet content of T lymphocytes was measured by flow cytometry using the BioTracker 488 Green Lipid Dye (SCT120, Millipore-Sigma, Burlington, MA) as described by the manufacturer. Briefly, T cells were collected from culture plates by centrifugation 300 x g for 10 minutes to remove media and then transferred to flow cytometry tubes in 1.0 mL PBS. Cells were washed with PBS and centrifuged at 300 x g for 10 minutes to remove the wash buffer. The cells were resuspended in PBS (100 µL) and BioTracker 488 Green Lipid Dye (excitation λ = 427 nm, emission λ = 585 nm) (5 µL) was added. Cells were vortexed gently and incubated at 37°C for 30 min in the dark. The cells were washed with PBS and collected by centrifugation at 400 x g for 5 min and resuspended in 100 µL staining buffer (BD Biosciences) and incubated with anti-CD3 (V550 conjugate, excitation λ = 403 nm, emission λ = 454 nm, clone UCHT1) plus anti-CD69 (phycoerythrin-cyanine 7 conjugate, excitation λ = 565 nm, emission λ = 774 nm, clone FN50; BD Biosciences, UK) antibodies (5 µL each) at 4°C for 30 min in the dark, washed with PBS, resuspended in 500 µL of PBS and analysed using an Attune NxT flow cytometer (Invitrogen, Massachusetts, USA). Raw data were analysed using FlowJo 10.8.1. (BD Biosciences) (**Supplementary Figure 2**).

### Statistical analyses

Statistical analyses were carried out using IBM SPSS Statistics for Windows, Version 27.0.

(Armonk, NY: IBM Corp). The data were assessed for normality by the Kolmogorov Smirnov test. Normally distributed data are shown as mean ± SEM, while data that did not follow a normal distribution are reported as median (range). Pairwise comparisons of were by Student’s t test for normally distributed data, or by the Mann-Whitney U test for non-normally distributed data. Statistical testing of the interaction between age and T cell activation status (activation state*life stage) was by 2-way ANOVA with Tukey’s *post hoc* correction for multiple comparisons. The threshold of statistical significance was set at p < 0.05. Comparisons between non-normally distributed data sets were by the Kruskal -Wallis Signed Rank Test, with *post hoc* pairwise testing using the Mann-Whitney U test. The relationships between data sets were tested by linear regression analysis.

## Results

### Participant characteristics

The median ages of the adult and senior participants were significantly different (p < 0.001). There were no other statistically significant differences in the general characteristics of the participants between these life stages.

### The effect of life stage and activation on CD3^+^ T lymphocyte fatty acid composition

There was a significant effect of life stage, but not activation status, on the amount of total fatty acids in T lymphocytes (**Table 1**). The amount of total fatty acids in T cells from seniors was greater than in T lymphocytes from adults, but less than in those from umbilical cord blood irrespective of activation state.

**Table 1:** Effect of life stage on activation-associated changes in the fatty acid composition of T lymphocytes.

	Fatty acid composition by life stage and activation state													
	Fetal			Adults				Seniors				ANOVA		
n	8			10				6				(P)		
	U	St		U		St		U		St		LS	AS	LS*AS
Total amounts of fatty acid classes (nanomoles/10^6^ cells)														
Total SFA	34.5 ± 4.8^a^	39.9 ± 5.5^a^		6.7 ± 0.5^b^		8.7 ± 1.2^b^		21.8 ± 7.0^c^		16.2 ± 2.0^c^		<0.0001	0.83	0.39
Total MUFA	6.5 ± 0.6^a^	14.1± 2.5^b^		2.0 ± 0.2^c^		3.6 ± 0.4^c^		3.2± 0.9^c^		3.8 ± 1.1^c^		<0.0001	0.002	0.012
Total ω-6	6.8 ± 0.6^a^	15.1± 2.4^b^		3.1 ± 0.1^c^		4.6 ± 0.3^c^		2.7± 0.8^c^		3.6 ± 1.1^c^		<0.0001	<0.0001	0.003
Total ω-3	1.0 ± 0.2^a^	1.5 ± 0.2^a^		0.5 ± 0.1^b^		0.7 ± 0.1^b^		2.1 ± 0.3^c^		2.2 ± 0.3^c^		<0.0001	0.84	0.39
Total FA	49.4 ± 5.3^a^	71.5 ± 10.3^a^		12.5 ± 0.7^b^		17.9 ± 2.0^b^		29.9 ± 6.7^c^		26.0 ± 4.1^c^		< 0.0001	0.089	0.081
Proportions of fatty acids (mol. % total fatty acids)														
14:0	19.3 ± 2.3^a^	12.9 ± 1.6^a^		3.6 ± 0.5^b^		3.3 ± 0.5^b^		1.6 ± 0.4^b^		2.6 ± 1.5^c^		< 0.0001	0.08	0.21
16:0	28.4 ± 0.9^a^	25.5 ± 0.6 ^a^		29.9 ± 0.7^a^		28.4 ± 0.5^a^		34.2 ± 2.4^b^		32.2 ± 1.6^b^		< 0.0001	0.06	0.8
18:0	18.3 ± 0.9^a^	15.3 ± 0.7^b^		21.9 ± 0.6^c^		18.2 ± 0.5^a^		32.8 ± 4.2^d^		29.1 ± 3.8^d^		< 0.0001	0.027	0.97
20:0	0.5 ± 0.1^a^	0.4 ± 0.1^a^		0.3 ± < 0.1^ab^		0.3 ± < 0.1^ab^		0.2 ± <0.1^b^		0.2 ± < 0.1^b^		0.004	0.79	0.89
16:1ω-7	1.1 ± 0.1	1.3 ± < 0.1		0.9 ± 0.1		1.1 ± < 0.1		0.7 ± 0.2		0.9 ± 0.2		0.07	0.08	0.89
18:1ω-9	11.7 ± 1.0^a^	16.8 ± 0.7^b^		12.1 ± 0.5^a^		14.6 ± 0.3^b^		9.8 ± 2.4^a^		11.3 ± 1.7^a^		0.006	0.001	0.23
18:1ω-7	1.4 ± 0.2^a^	1.6 ± 0.1^b^		1.4 ± 0.1^a^		1.5 ± < 0.1^b^		1.1 ± 0.2^c^		1.2 ± 0.1^c^		0.04	0.35	0.75
20:1ω-9	0.4 ± 0.1	0.4 ± 0.1		0.4 ± < 0.1		0.7 ± 0.3		0.3 ± 0.1		0.2 ± < 0.1		0.23	0.70	0.49
18:2ω-6	9.2 ± 0.9^a^	14.4 ± 0.7^b^		12.6 ± 0.3^a^		16.1 ± 0.5^b^		8.3 ± 2.3^a^		11.0 ± 2.1^b^		< 0.0001	< 0.0001	0.52
20:2ω-6	0.3 ± < 0.1^a^	0.3 ± <0.1^a^		1.1 ± 0.1^b^		1.3 ± 0.1^b^		0.3 ± <0.1^a^		0.3 ± < 0.1^a^		< 0.0001	0.47	0.62
20:3ω-6	0.9 ± 0.2^a^	1.3 ± 0.1^a^		1.4 ± 0.1^b^		1.5 ± 0.1^b^		0.8 ± 0.1^a^		0.9 ± 0.1^a^		< 0.0001	0.06	0.35
20:4ω-6	4.9 ± 0.7^a^	6.1 ± 0.5^a^		10.1 ± 0.5^b^		8.7 ± 0.4^ab^		6.1 ± 1.1^a^		6.3 ± 0.7^a^		< 0.0001	0.99	0.1
22:4ω-6	0.9 ± 0.1	1.1 ± 0.1		0.3 ± < 0.1		0.3 ± < 0.1		0.5 ± 0.1		0.6 ± 0.1		0.85	0.17	0.48
18:3ω-3	0.9 ± 0.1^a^	0.9 ± < 0.1^a^		1.4 ± 0.2^b^		1.7 ± 0.2^b^		1.0 ± 0.2^a^		1.0 ± 0.1^a^		< 0.0001	0.21	0.52
20:3ω-3	0.3 ± 0.1	0.3 ± 0.1		0.2 ± < 0.1		0.2 ± < 0.1		0.3 ± < 0.1		0.2 ± < 0.1		0.17	0.77	0.67
20:5ω-3	0.1 ± < 0.1	0.1 ± < 0.1		0.1 ± < 0.1		0.0 ± < 0.1		0.2 ± < 0.1		0.2 ± < 0.1		< 0.0001	0.65	0.87
22:5ω-3	0.3 ± 0.1^a^	0.1 ± < 0.1^a^		0.9 ± 0.1^b^		0.8 ± < 0.1^b^		0.6 ± 0.1^b^		0.6 ± 0.1^b^		< 0.0001	0.29	0.31
22:6ω-3	0.8 ± 0.1	0.8 ± 0.1		1.0 ± 0.1		0.8 ± < 0.1		0.8 ± 0.1		0.9 ± 0.1		0.73	0.54	0.24

Values are mean ± SEM. Statistical analysis was by 2-Way ANOVA with life stage (LS) and activation state (AS) as fixed factors with Tukey’s post hoc test. Means with different superscripts were significantly different (p < 0.05). FA, fatty acids; MUFA, monounsaturated fatty acids; St, stimulated cells; SFA, saturated fatty acids; U, unstimulated cells.

There was a significant single factor effect of life stage but not activation status, on the total amount of SFAs (**Table 1**) such that the amount of total SFAs in T cells from seniors was greater than T lymphocytes from adults, but less than in those from umbilical cord blood irrespective of activation state. T lymphocytes from seniors contained less 14:0 and 20:0, but more 16:0 and 18:0 than T cells from umbilical cord blood or adults (**Table 2**).

**Table 2:** Amounts of [^13^C]-labelled fatty acids in unstimulated and mitogen-stimulated T lymphocytes.

	Amounts of labelled fatty acids (picomoles/10^6^ cells)								
	Fetal		Adults		Seniors		ANOVA		
n	8		10		6		(P)		
	U	St	U	St	U	St	LS	A	LS*A
18:3ω-3	5.9 ± 1.0^a^	14.6 ± 2.4^b^	14.3 ± 2.5^b^	19.0 ± 4.0^b^	6.1 ± 1.9^a^	7.5 ± 1.7^a^	0.002	0.021	0.4
20:3ω-3	0.2 ± 0.1	0.3 ± 0.1	0.1 ± 0.1	0.1 ± 0.0	0.2 ± 0.1	0.3 ± 0.1	0.4	0.5	0.4
20:5ω-3	0.1 ± 0.0	0.1 ± 0.0	0.1 ± 0.0	0.3 ± 0.2	0.1 ± 0.0	0.0 ± 0.0	0.2	0.3	0.3
22:5ω-3	ND	ND	ND	ND	ND	ND			
22:6ω-3	ND	ND	ND	ND	ND	ND			
Total ω-3 PUFA‡	0.3 ± 0.1	0.4 ± 0.1	0.2 ± 0.1	0.4 ± 0.2	0.3 ± 0.1	0.3 ± 0.1	0.8	0.3	0.7
16:0	5.2 ± 0.6^a^	6.7 ± 1.2^a^	1.7 ± 0.2^b^	1.7 ± 0.2^b^	3.3 ± 1.0^ab^	3.0 ± 0.5^ab^	<0.001	0.5	0.4
16:1ω-7	ND	ND	ND	ND	ND	ND			
18:0	3.7 ± 0.6^a^	4 ± 0.7^a^	1.3 ± 0.1^b^	1.3 ± 0.1^b^	3.7 ± 1.3^a^	2.6 ± 0.4^a^	<0.001	0.6	0.6
18:1ω-9	2.7 ± 0.9^a^	4 ± 0.8^a^	1.0 ± 0.1^b^	1.0 ± 0.1^b^	0.8 ± 0.2^b^	1.4 ± 0.3^ab^	<0.001	0.1	1.0
Total SFA+MUFA	11.6 ± 1.9^a^	14.9 ± 2.6^a^	4.0 ± 0.4^b^	3.9 ± 0.4^b^	7.78 ± 2.3^ab^	7.1 ± 1.0^ab^	<0.001	0.6	0.5

Values are mean ± SEM. Statistical analysis was by 2-Way ANOVA with life stage (LS) and activation state (A) as fixed factors with Tukey’s post hoc test. Means with different superscripts were significantly different (p < 0.05). Rows without superscripts indicate that there were no significant differences between any of the means for that row. ND, not detected; MUFA, monounsaturated fatty acids; PUFA, polyunsaturated fatty acids; St, stimulated cells; SFA, saturated fatty acids; U, unstimulated cells. ‡Sum of 20:3ω-3, 20:5ω-3, 22:5ω-3 and 22:6ω-3 derived from interconversion of 18ω-3.

There were significant single factor effects of life stage and activation state, and a significant interaction effect of activation state*life stage on the amount of total MUFAs in T lymphocytes (**Table 1**) such that the total amount of MUFAs in T cells from seniors was greater than in T lymphocytes from adults irrespective of activation state (**Table 1**). Stimulated fetal cells contained 2.2-fold more MUFAs than unstimulated fetal cells, and 3.7- to 7-fold more total MUFAs than cells from adult or senior participants irrespective of activation state (**Table 1**). The proportions of 18:1ω-9 and 18:1ω-7 were lower in T cells from seniors, than in those from adults or umbilical cord blood. Con. A stimulation significantly increased the proportion of 18:1ω-9 in fetal CD3^+^ T cells and in CD3^+^ T lymphocytes from adults, but not seniors. The proportions of 16:1ω-7 and 20:1ω-9 did not differ between life stages or T cell activation state.

There were significant single factor effects of life stage and activation state, and a significant interaction effect of activation state*life stage on the amount of total ω-6 PUFAs in T lymphocytes (**Table 1**). The total amount of ω-6 PUFAs in T cells from senior participants was less than in either fetal or adult cells of corresponding activation state (**Table 1**). There were significant single factor effects of life stage and activation state, but no significant interaction effect on the proportion of 18:2ω-6. Con. A stimulation increased the proportion of 18:2ω-6 in cells from all life stages (**Table 1**). The proportion of 18:2ω-6 was lower in T cells from seniors than fetal cells or T cells from adults. T cells from adults contained significantly more 20:2ω-6, 20:3ω-6 and 20:4ω-6 than those from either umbilical cord blood or seniors, and there was no significant effect of activation on the proportions of these ω-6 PUFAs. In agreement with previous findings (22), 18:3ω-6 was not detected at any life stage.

There was a significant single factor effect of life stage, but not of activation state, and no significant interaction effect of activation state*life stage on the amount of total ω-3 PUFAs in T lymphocytes (**Table 1**). The total amount of ω-3 PUFAs in T cells from senior participants was greater than in either fetal or adult cells of corresponding activation state (**Table 1**). The proportions of 18:3ω-3 and 22:5ω-3 in cells from adult participants were greater than in T lymphocytes from either umbilical cord blood or senior participants, irrespective of activation state. There were no significant effects of life stage or activation state on the proportions of 20:3ω-3, 20:3ω-3 or 22:ω-6 (**Table 1**). The proportion of 20:5ω-3 was greater in T cells from seniors, while fetal T cells contained less 22:5ω-3 than cells from adults (**Table 1**).

### The effect of life stage and activation on T lymphocyte ω-3 polyunsaturated fatty acid synthesis

[^13^C]-Enrichment was detected in 18:3ω-3, 20:3ω-3 and 20:5ω-3, but not in 22:5ω-3 or 22:6ω-3 (**Table 2**). There were significant single factor effects of life stage and activation state, but there was no significant life stage*activation state interaction effect on T cell [^13^C]18:3ω-3 content (**Table 2**). [^13^C]18:3ω-3 content of unstimulated cells after 48 hours in culture was significantly (approximately 2.4-fold) greater in T cells from adults compared to fetal T cells or T cells from seniors. The amount of [^13^C]18:3ω-3 in stimulated fetal T cells was 2.5-fold greater than in unstimulated cells, while there was no significant effect of stimulation on the amount of [^13^C]18:3ω-3 in cells from adults or seniors (**Table 2**). There were no significant single factor effects of age or activation, and no significant life stage*activation interaction effect on T cell [^13^C]20:3ω-3 or 20:5ω-3 contents (**Table 2**).

### [^13^C]-Enrichment of saturated and monounsaturated fatty acids

[^13^C]-Enrichment of 16:0, 18:0 and 18:1ω-9 was detected in unstimulated and stimulated T cells from all life stages after 48 hours of culture (**Table 3**). There was a significant effect of age, but not activation state, on the amounts of labelled 16:0, 18:0 and 18:1ω-9. There was between 3- to 4-fold more [^13^C]16:0, [^13^C]18:0 and [^13^C]18:1ω-9 in fetal cells compared to cells from adults. There were similar amounts of [^13^C]16:0 and [^13^C]18:0 in cells from seniors to fetal cells, while the amount of [^13^C]18:1ω-9 was between 65% and 70% lower in cells from senior participants than in fetal cells (**Table 3**). No [^13^C]-enrichment of 16:1ω-7 was detected. The sum of [^13^C]-labelled 16:0, 18:0 and 18:1ω-9 was used as a proxy measure for partitioning of 18:3ω-3 towards fatty acid 𝛽-oxidation and fatty acid synthesis *de novo*. There was a significant effect of life stage, but not cell activation, on the sum of the amounts of [^13^C]16:0, [^13^C]18:0 plus [^13^C]18:1ω-9 (**Table 2**). The sum of [^13^C]-labelled SFAs and MUFAs was significantly greater in fetal T lymphocytes than in T cells from adults or seniors. There was no significant difference in the sum of labelled SFAs plus MUFAs between cells from adult and senior participants (**Table 2**). The proportion of total SFAs plus total MUFAs accounted for by total [^13^C]SFAs plus MUFAs were fetal 0.03%, adult 0.03% and Seniors 0.04%.

**Table 3:** Amounts of [^13^C]18: 3ω-3-derived oxylipins in unstimulated and mitogen-stimulated T lymphocytes.

	Amount of oxylipin (picomoles/10^6^ cells)								
	Fetal		Adults		Seniors		ANOVA		
n	8		8		8		(P)		
	U	St	U	St	U	St	LS	A	LS*A
9-HOTrE	99.7 ± 14.0^a^	111.0 ± 17.9^a^	31.7 ± 1.3^b^	33.1 ± 2.0^b^	29.1 ± 5.0^b^	30.3 ± 4.5^b^	< 0.001	0.6	0.8
13-HOTrE	33.5 ± 4.2^a^	38.5 ± 5.3^a^	15.0 ± 0.4^b^	17.8 ± 1.0^b^	12.9 ± 1.5^b^	12.8 ± 1.2^b^	< 0.001	0.4	0.7
9,10-DiHODE	13.6 ± 1.6^a^	17.68 ± 2.2^b^	12.4 ± 1.3^a^	15.4 ± 0.7^ab^	6.7 ± 0.7^c^	6.6 ± 0.8^c^	< 0.001	0.04	0.97
12,13-DiHODE	9.7 ± 0.8^a^	13.2 ± 1.6^b^	9.4 ± 1.3^a^	11.4 ± 0.6^a^	5.4 ± 0.6^c^	5.5 ± 0.7c	< 0.001	0.04	0.2
15,16-DiHODE	9.1 ± 0.7^a^	11.1 ± 0.1^a^	10.1 ± 0.6^a^	11.4 ± 0.9^a^	4.5 ± 0.4^b^	4.4 ± 0.4^b^	< 0.001	0.08	0.4
Total‡	165.6 ± 20.9^a^	191.5 ± 26.8^a^	79.7 ± 2.8^b^	89.1 ± 4.5^b^	58.7 ± 6.6^c^	59.5 ± 6.6^c^	< 0.001	0.4	0.7

Values are mean ± SEM. Statistical analysis was by 2-Way ANOVA with life stage (LS) and activation state (A) as fixed factors. The threshold of statistical significance was p < 0.05. Means with different superscripts were significantly different by 2-way ANOVA with Tukey’s post hoc` test. Rows without superscripts indicate that there were no significant differences between any of the means for that row. ‡Sum of all detected [^13^C]-labelled hydroxy- and dihydroxy- octadecaenoic acids. St, stimulated cells; U, unstimulated cells.

### The effect of age and activation on the concentrations of ω-3 oxylipins in T cell culture supernatants

Five [^13^C]-labelled oxylipin species derived from 18:3ω-3 were detected in cell culture supernatants of unstimulated and mitogen-stimulated T lymphocytes at all life stages (**Table 3**). 9-HOTrE was the principal 18:3ω-3-derived oxylipin in supernatants from T cell cultures in all life stages. The amount of 9-HOTrE was approximately 3-fold greater in supernatants of fetal cell cultures than from cultures of cells from adults or seniors participants, irrespective of activation state (**Table 3**). There was a significant effect of life stage on the amount of all five [^13^C]-labelled oxylipin species and a significant effect of activation state on the amounts of [^13^C]-9,10-DiHODE and [^13^C]-12,13-DIHODE, but not on the other labelled oxylipin species (**Table 3**). There was no significant life stage*activation state interaction effect on the amount of any of the five labelled oxylipins detected in culture supernatants after 48 hours (**Table 3**). The amounts of individual and total oxylipin synthesised by fetal cells tended to be significantly greater than those produced by T cells from adults or seniors (**Table 3**).

### Comparison of the relative amounts of 18:3ω-3-derived PUFAs, oxylipins and SFAs plus MUFAs

**Figure 1:**
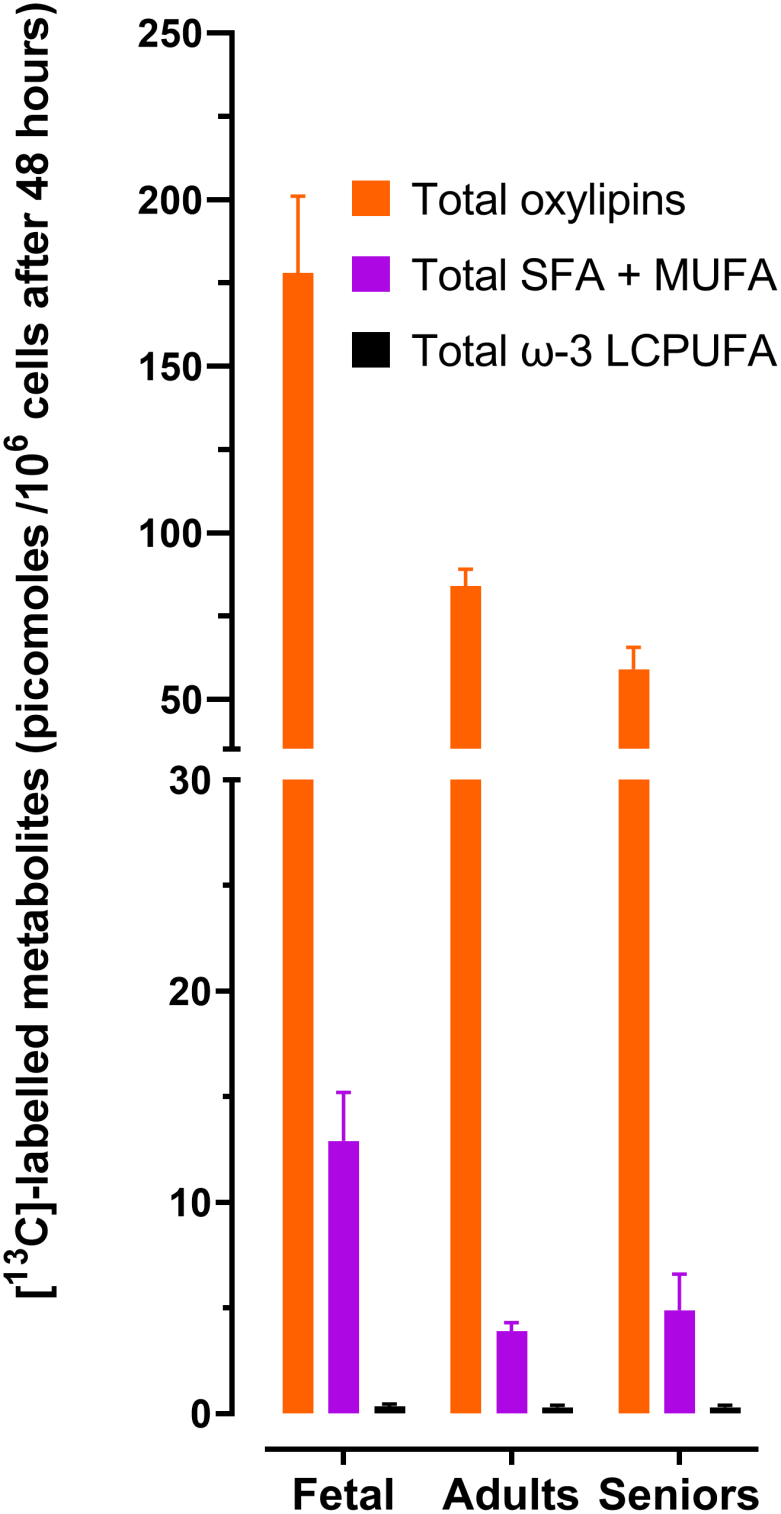
Summary of the effect of life stage on the amounts total [^13^C]-labelled 18:3ω-3 – derived metabolites in CD3^+^ T lymphocytes. Values are the mean ± SEM of each [^13^C]-labelled fatty acid class in unstimulated plus stimulated T cells according to life stage. Data and statistical comparisons are shown in **Tables 2**, **3**. LCPUFA, long-chain polyunsaturated fatty acids; MUFA, monounsaturated fatty acids; SFA, saturated fatty acids. Total oxylipins were the sum of [^13^C]-9-HOTrE, [^13^C]-13-HOTrE, [^13^C]-9,10-DIHODE, [^13^C]-12,13-DiHODE, plus [^13^C]-15,16-DiHODE. Total LCPUFA were the sum of [^13^C]20:3ω-3,[^13^C]20:5ω-3, [^13^C]22:5ω-3 plus [^13^C]22:6ω-3. Total SFA + MUFA were the sum of [^13^C]16:0 + [^13^C]18:0 + [^13^C]18:1ω-9.

Comparison of the mean of the total amounts of labelled oxylipins, SFAs plus MUFAs and ω-3 PUFAs showed that the rank order of relative metabolic partitioning of 18:3ω-3 in T lymphocytes from all life stages was oxylipin synthesis and secretion > SFA plus MUFA synthesis > conversion to longer chain ω-3 PUFAs irrespective of activation state (**Tables 2**, **3** and **Figure 1**). In fetal cells, oxylipin synthesis was 13-fold greater than SFA+MUFA synthesis, which was 37-fold greater than PUFA synthesis. In cells from adults, oxylipin synthesis was 22-fold greater than SFA+MUFA synthesis, which was 13-fold greater than PUFA synthesis. In cells from seniors, oxylipin synthesis was 12-fold greater than SFA+MUFA synthesis, which was 16-fold greater than PUFA synthesis (**Tables 2**, **3** and **Figure 1**).

### The effect of life stage on the lipid droplet content of total CD3^+^ lymphocytes

**Figure 2:**
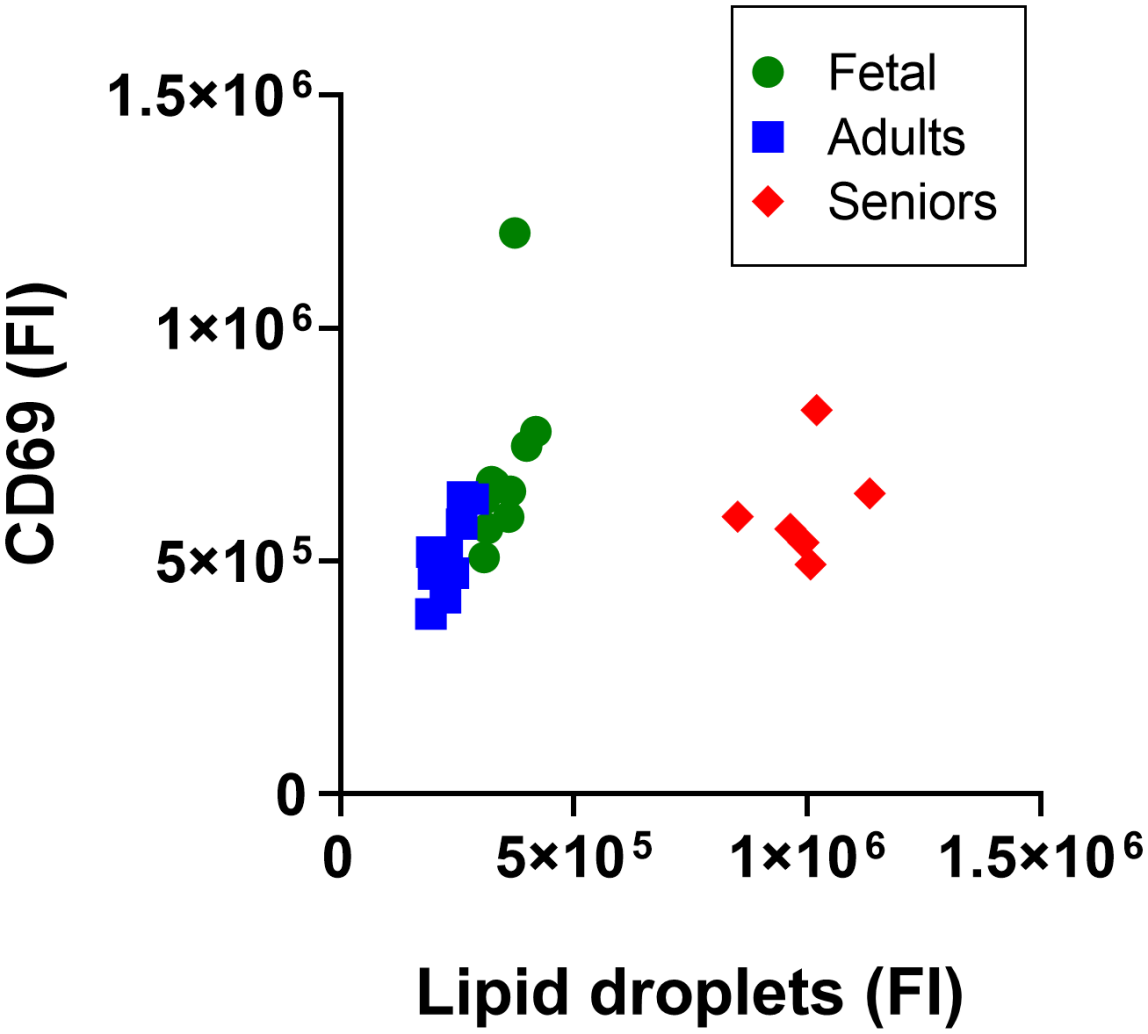
Analysis of the lipid droplet content of activated CD3^+^ T lymphocytes according to life stage. Data are median fluorescence intensity by flow cytometry of CD3^+^ T lymphocytes from individual participants; Fetal T cells from umbilical cord, and adults and seniors. a.u., absorbance units.

Plotting the fluorescence index from lipid droplets against that from CD69 expression, showed that CD3^+^ T cells from seniors clustered separately from cells from adults or umbilical cord (**Figure 2**), while the distributions of fetal and adult cells overlapped. Combined analysis of adult and fetal cells showed that the lipid droplet content and CD69 expression were associated positively. There was a statistically significant positive association between lipid droplet content and CD69 expression for cells from adults (r^2^ = 0.62, p = 0.007, degrees of freedom (*df*) 1.8, F = 13.22, n = 10) such that variation in lipid droplet content predicted 62% of the variation in the cell surface expression of CD69 (**Figure 2**) There was no statistically significant association between lipid droplet content and CD69 expression for fetal cells (r^2^ = 0.26, p = 0.13, n = 10) or cells from seniors (r^2^ = 0.06, p = 0.65, n = 7).

## Discussion

The present findings show that the fatty acid composition and metabolism of 18:3ω-3 in human T lymphocytes differs between life stages, particularly in the relative partitioning between synthesis of longer chain PUFAs and production of 18-carbon oxylipins, while mitogen activation exerted a minor effect on these pathways. Furthermore, the lipid droplet content of CD3^+^ T cells differed according to life stage.

Life stage influenced the total amounts of SFAs, MUFAs and ω-3 PUFAs as well as ω-6 PUFAs such that the amounts of these fatty acids were greater in quiescent fetal T cells than in cells from adults or seniors. However, the effect of life stage on mitogen-induced changes in T cell fatty acid composition was restricted to the total amounts of MUFAs and ω-6 PUFAs.

One previous study found that mitogen stimulation of human T cells increases the proportions of 18:1ω-9, 22:5ω-3 and 22:6ω-3, and decreases the proportions of 20:1ω-9 and 20:2ω-6 which were associated with altered membrane fluidity (40, 41). Others have reported that Con. A stimulation increased the amounts of 18:3ω-3 and 18:2ω-6 and induced selective changes in some MUFA and PUFA species with chain length greater than 20 carbon atoms in T cell total lipids from adults (22). The activation-associated changes in fatty acid composition in cells from adult participants in the present study were in general agreement with previous observations (21, 22, 42, 43).

Lipidomic analysis of human umbilical cord CD4^+^ T cells found that activation-induced differential changes in the proportions of individual phosphatidylcholine and phosphatidylethanolamine molecular species, particularly in the amounts of unsaturated species (44), which involve modifications to phospholipid acyl remodelling processes (42, 44, 45). The specificity of phospholipid biosynthesis, including acyl remodelling processes, is regulated by the stage of development in lung and liver tissue (46, 47), and by endocrine factors in liver (48). If it is assumed that the fatty acid composition of total T cell lipids reflects primarily the composition of membrane phospholipids, although non-membrane structures such as lipid droplets may also be included, one interpretation of the present findings is the specificity of phospholipid metabolism changes across the life course. If so, since the composition of membrane phospholipids can influence the activity of integral proteins (49) *via* the homeoviscous adaptation of the lipid bilayer (41, 49, 50) and the production of lipid mediators (24, 51, 52), such life course changes may contribute to characteristic patterns of immune function of different life stages (27, 30).

Whole body and tissue-specific recycling of carbon from 18:3ω-3 into 16:0, 16:1ω-7, 18:0, 18:1ω-9 has been reported in rats (52–55), rhesus macaques (56) and humans (57). The total amount of [^13^C]-SFAs plus [^13^C]-MUFAs was significantly greater in fetal T cells incubated with [^13^C]18:3ω-3 compared to cells from seniors > adults. Since the pattern of [^13^C]-SFA plus [^13^C]-MUFA synthesis between life stages was similar to that of the total fatty acid content, one possible explanation is that the higher total fatty acid content of fetal T cells compared to cells from older life stages represents greater partitioning of newly assimilated fatty acid towards β-oxidation and recycling of carbon atoms by fatty acid synthesis *de novo* which suggests T cell fatty acid β-oxidation declines with increasing age possibly reflecting changes in proportions of T cell subsets with different metabolic requirements (19, 58, 59). The precise metabolic function of carbon recycling from 18:3ω-3 into SFAs and MUFAs is not known. It is important for cholesterol synthesis in neonatal rat brain (53), possibly as a substrate for membrane synthesis, and tracked with developmental changes in fatty acid composition. The present findings show that recycling of carbon from [^13^C]18:3ω-3 into SFAs and MUFAs tracked through life course changes in the total SFA plus MUFA contents of T lymphocytes. However, carbon recycling only accounted for less than 0.05% of the total amount of SFAs plus MUFAs in all life stages, and, therefore, this mechanism may not be a quantitatively important source of these fatty acids in T lymphocytes, unless turnover of the T cell SFA plus MUFA pool is rapid compared to synthesis by fatty acid synthesis *de novo*. Whether carbon recycling contributes significantly to cholesterol synthesis in T cells is not known. One further possibility is that β-oxidation of 18:3ω-3 and carbon recycling into SFAs represents a mechanism to limit the accumulation of fatty acids that are susceptible to peroxidation.

It has been suggested that partitioning between the synthesis of PUFAs and hydroxyoctadecadi- or tri-enoic acids is a branch point in EFA metabolism and that 9- and 13-HOTrE synthesis is constitutive and independent of the activation state of the cells and may represent an immuno-homeostatic process (22). Five 18:3ω-3-derived oxylipin species were identified in the present study which differed in amount between life stages, such that culture supernatants of fetal cells contained the greatest amounts of individual and total [^13^C]-labelled oxylipins comprised of 18 carbon atoms, followed by supernatants of T cells from the Nod-like receptor family pyrin domain containing-3 (NLRP3) inflammasome complex *via* a PPARγ –dependent mechanism in murine peritoneal macrophages (60). If this process occurs in the human immune system, enhanced constitutive synthesis and secretion of 13-HOTrE by fetal CD3^+^ T lymphocytes could represent one mechanism in fetal immune tolerance and the decline in production later in the life course may be a contributory factor in inflammation associated with immunosenescence. This suggestion is supported by he observation that the 18:3ω-3 content of neutrophils increases during pregnancy which has been suggested as a possible mechanism in maternal immune tolerance (61).

Conversion of [^13^C]18:3ω-3 to longer chain PUFAs was restricted to [^13^C]20:3ω-3 and [^13^C]20:5ω-3. This is consistent with previous findings that show the first reaction in the T lymphocyte PUFA synthesis pathway is carbon chain elongation, possibly catalysed by elongase-5, followed by Δ8 and Δ5 desaturation (21–23) with no detectable synthesis of PUFAs longer than 20 carbon atoms due to the absence of elongase-2 expression (21–23). There were no significant differences between life stages in the capacity of T cells to convert [^13^C]18:3ω-3 to longer chain PUFAs. This suggests that if partitioning between oxylipin and PUFA synthesis is a branch point in T lymphocyte essential fatty acid metabolism, the mechanism by which the life stage modifies the capacity for oxylipin synthesis does not involve a simple reciprocal relationship with PUFA synthesis.

The lipid droplet content of CD3^+^ T cells was greater in seniors than in fetal or adult cells which is consistent with previous findings that CD8^+^ T cells from seniors exhibited greater fatty acid accumulation than cells from younger individuals (27). This was accompanied by impaired proliferation and increased expression of apoptotic markers consistent with impaired immune response in seniors (27). The present findings from analysis of total CD3^+^ T cells do not exclude the possibility of any effects of lipid droplets T cell function may differ between T cell subsets. Nevertheless, these findings suggest that the accumulation of lipid droplets in T cells from healthy adults enhances processes associated with cell proliferation which is consistent with the regulatory functions of lipid droplets in T lymphocytes (28, 29). The absence of a statistical association between the lipid droplet content and CD69 expression in fetal T cells and T lymphocytes from seniors implies that the specific function of lipid droplets in T cells from adults may be of lesser importance for activation in fetal T cells or T lymphocytes from seniors. There was an apparent disconnect between the relative amounts of total fatty acids between life stages and the lipid droplet content of activated T cells in that fetal cells had the highest amount of total fatty acids of the three groups while T cells from seniors had the greatest lipid droplet content. One possible explanation is that fetal cells contained more fatty acids associated with cell membranes membranes. Alternatively this apparent disconnent may have been due to differences in the methods used to measure fatty acids and lipid droplets. Previous analyses of the lipid droplet content of T lymphocytes used similar semi-quantitative methods to those used here (27) and previous studies that quantified the amount of individual lipid classes associated with lipid droplets did not normalise the lipid mass to the number of cells (62, 63), and the proportional contribution of lipid droplets to the total fatty acid content of mammalian T cells is not known. Although triacylglycerol is a major component of lipid droplets, it is possible that because of the notably smaller size of lipid droplets compared to T lymphocytes (63), the total mass of fatty acids incorporated into lipid droplets may be relatively small compared to the fatty acid content of cell membrane phospholipids and hence any variation in lipid droplet content may be masked by the amount of fatty acid incorporated into membrane phospholipids, particularly in stimulated cells undergoing blastic transformation.

Overall, the present findings show that fatty acid metabolism in CD3^+^ lymphocytes differs between life stages in a manner which suggests a possible causal role in the changes in T cell function that occur over the life course (27, 30, 40). These findings have implications for understanding differences in immune function between individuals in their response to vaccines, susceptibility to infection and risk of chronic non-communicable inflammatory diseases.

## Data availability statement

The raw data supporting the conclusions of this article will be made available by the authors, without undue reservation.

## Ethics statement

The studies involving human participants were reviewed and approved by The study was reviewed and approved by the East of England - Cambridge Central Research Ethics Committee (approval number 19/EE/0096) and all donors gave written informed consent. The purchase and use of adult and fetal primary leukocytes that were collected in accordance with Local, State and Federal U.S. requirements by StemCell Technologies, (Vancouver BC, Canada), was reviewed and approved by the University of Southampton Faculty of Medicine Ethics Review Committee (submission I.D.S 49658 and 58050.A1). Written informed consent to participate in this study was provided by the participants’ legal guardian/next of kin.

## Author contributions

GB, BF, PC, EM, and KL conceived and designed the study. JG, AW, and NI conducted the experiments and analysed the data with GB. GB wrote the first draft of the manuscript. All authors contributed to the article and approved the submitted version.
