# Supplementary material for: Fatty acid composition and metabolic partitioning of α-linolenic acid are contingent on life stage in human CD3+ T lymphocytes
Source: Front Immunol. 2022 Dec 13;13:1079642. doi: 10.3389/fimmu.2022.1079642 (PMC9792684; doi:10.3389/fimmu.2022.1079642)
Supplement: Supplementary file 1 [file DataSheet_1.pdf]

Supplementary Online Material

Fatty acid composition and metabolic partitioning of α-linolenic acid are contingent on life stage in human CD3^{+} T lymphocytes

Annette. L. West, Johanna von Gerichten, Nicola A. Irvine, Elizabeth A. Miles, Karen A. Lillycrop, Philip C. Calder, Barbara A. Fielding and Graham C. Burdge

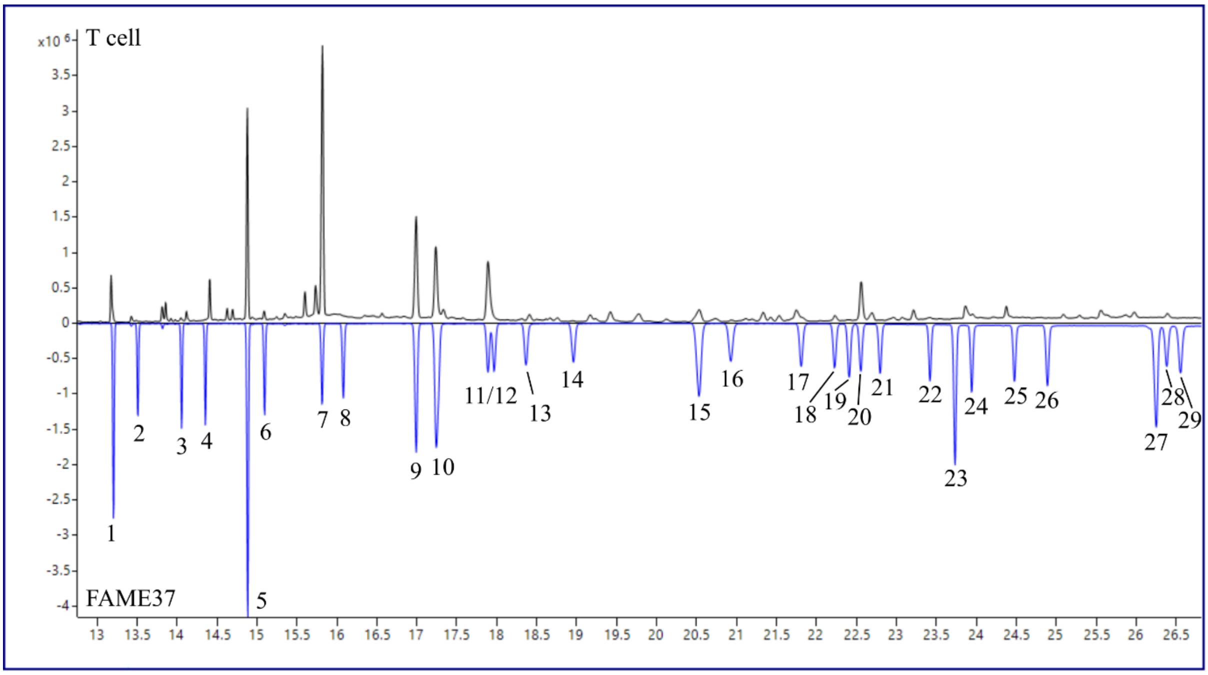

Supplementary Figure 1. Total ion Chromatogram of fatty acid methyl esters from resting 48h cultured CD3^{+} T cells (upper panel, black) and of FAMEs37 standard (lower panel, blue; Supelco CRM47885). [1] C14:0, [2] C14:1, [3] C15:0, [4] C15:1, [5] C16:0, [6] C16:1, [7] C17:0, [8] C17:1, [9] C18:0, [10] C18:1n9, [11] C18:2ω-6*cis*, [12] C18:2ω-6*trans*, [13] C18:3ω-6, [14] C18:3ω-3, [15] C20:0, [16] C20:1ω9, [17] C20:2ω-6, [18] C20:3ω-6, [19] C21:0, [20] C20:4ω-6, [21] C20:3ω-3, [22] C 20:5ω-3, [23] C22:0, [24] C22:1n9, [25] C22:2ω-6 [26] C23:0, [27] C24:0, [28] C22:6ω-3, [29] C24:1ω-9. x-axis, time (minutes), y-axis 37FAMEs (abundance) T cells (minus abundance).

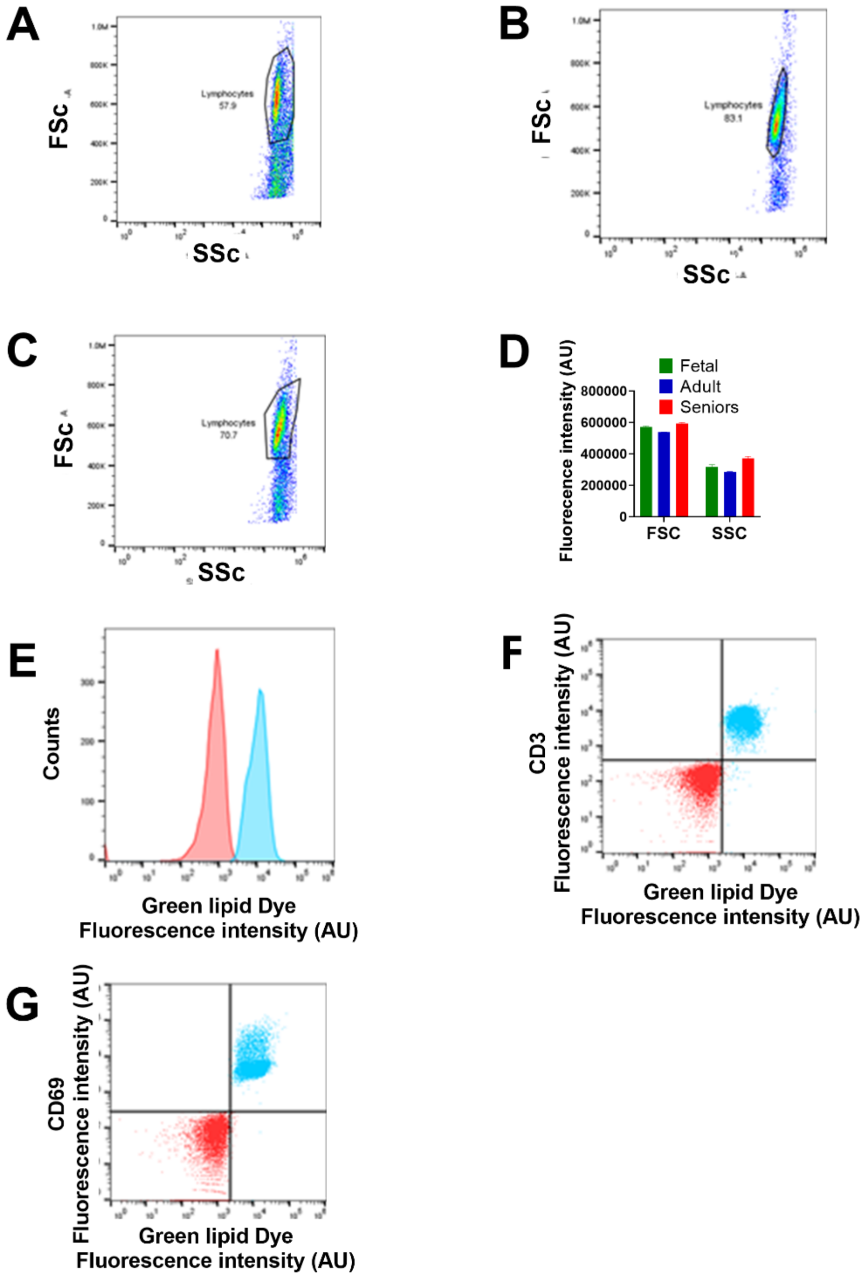

Supplementary Figure 2. Typical forward scatter (FSc) versus side scatter (SSc) plots of activated T cell preparations from (A) umbilical cord (fetal) (B) adults and (C) seniors. (D) A Summary of A B, C). Values are mean ±SEM geometric mean fluorescence intensity for n = 6 T cell cultures per life stage. Statistical analysis by one-way ANOVA with Tukey's *post hoc* correction for multiple comparisons showed no significant differences between life stages in either FSc or SSc. (E) A typical example of background (red) and positive staining with BioTracker 488 Green Lipid Dye (Blue positive stain, red, negative stain. (F) A Typical example of CD3 and BioTracker 488 Green Lipid Dye dual staining, (G) A Typical example of CD69 and BioTracker 488 Green Lipid Dye dual staining. Both F and G Show separation of fluorescence signals. AU, arbitrary units.
